# A perfect recipe for a corticospinal neuron

**DOI:** 10.7554/eLife.102425

**Published:** 2024-09-26

**Authors:** Alfonso Aguilera, Marta Nieto

**Affiliations:** 1 Department of Molecular and Cellular Biology, Centro Nacional de Biotecnología (CNB-CSIC) Consejo Superior de Investigaciones Científicas Madrid Spain

**Keywords:** reprogramming, corticospinal neurons, glial progenitors, neuroscience

## Abstract

A tailored cocktail of genes can reprogram a subset of progenitors to no longer produce glial cells and instead develop into neurons involved in motor control.

**Related research article** Ozkan A, Padmanabhan HK, Shipman SL, Azim E, Kumar P, Sadegh C, Basak AN, Macklis JD. 2024. Directed differentiation of functional corticospinal-like neurons from endogenous SOX6+/NG2+ cortical progenitors. *eLife* **13**:RP100340. doi: 10.7554/eLife.100340.

The skin, like many other tissues in our body, has a remarkable capacity to regenerate itself following an injury or during aging. Most regions in the brain and the spinal cord, however, cannot replace lost or damaged neurons. As a result, spinal cord injuries and neurodegenerative diseases, such as Alzheimer’s, have irreversible consequences.

During development, a group of progenitor cells give rise to the diverse set of neurons that form the brain, with each type of neuron having its own unique characteristics, residing in specific locations, and performing specialized functions. These progenitors disappear once the brain is built, thus preventing the generation of new neurons.

Discoveries over the past century indicate that it may be possible to replace lost or damaged neurons by reprogramming cells in the mature brain to take on a different fate. This is achieved by expressing so-called ‘reprogramming genes’ which instruct cells to differentiate into another cell type. However, the procedure is complex as it involves not only activating new genes, but also resetting the cell’s original identity. Therefore, both selecting the optimal set of reprogramming genes and the cells to reprogram are crucial to the success of the conversion method.

Current strategies for directed reprogramming have so far managed to generate neurons both in vitro and in vivo, but these neurons often exhibit immature or incomplete identities. This is because the reprogramming process does not fully replicate the molecular and environmental conditions that naturally give rise to the cells they aim to replace (Grande et al., 2013; Greig et al., 2013). Now, in eLife, Jeffrey Macklis (Harvard University) and co-workers – including Abdulkadir Ozkan and Hari Padmanabhan as joint first authors – report how they perfected two key aspects of the reprogramming procedure (Ozkan et al., 2024).

First, the team carefully selected which cells to reprogram. They chose progenitor cells in the cerebral cortex which typically produce oligodendrocytes – these are glial cells that, unlike neurons, are capable of proliferation and renewal throughout their lifespan. These glial progenitors are characterized by the expression of the protein NG2 (Zhu et al., 2008), and a subset has been shown to retain a dormant capacity to produce neurons (Belachew et al., 2003; Guo et al., 2010). Ozkan, Padmanabhan et al. found that this ability to generate neurons is blocked by the expression of SOX6, a protein that represses the gene *Neurogenin2* (*Neurog2*), which is responsible for the production of neurons in the embryo.

Second, the team developed a procedure to purify and culture the NG2 and SOX6 positive progenitors in a well-controlled in vitro environment (Figure 1B). Under these conditions, the morphology of the cells was similar to that of immature glia progenitors, and the pattern of genes they expressed reflected their undifferentiated state and potential capacity to generate various cell types (Figure 1C; Rivers et al., 2008).

**Figure 1.**
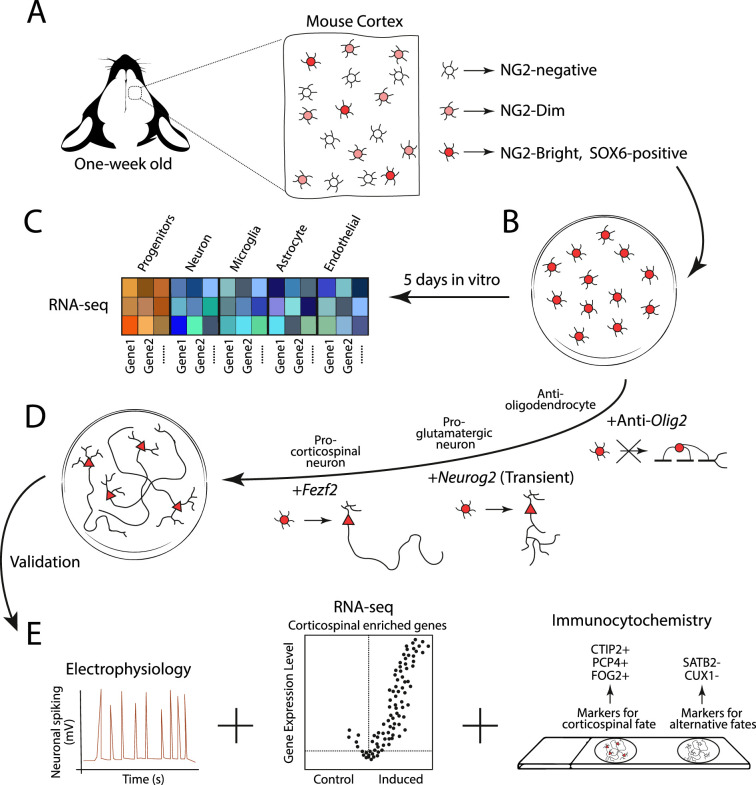
Experimental design for generating corticospinal neurons. (**A**) Glial progenitor cells in the cortex of one-week old mice express either high (NG2-Bright; dark red), medium (NG2-Dim; light red), or non-detectable (NG2-negative; white) levels of the protein NG2. The NG2-Bright cells also express the gene for the protein SOX6, which stops cells from producing neurons. (**B**) Ozkan, Padmanabhan et al. isolated and cultured the NG2 and SOX6 positive glial progenitors over five days and performed an RNA-seq experiment to see which genes they expressed. (**C**) This revealed that they express genes associated with undifferentiated identities (red and orange squares), but not genes associated with the mature cells of the brain, such as neurons, microglia, astrocytes and endothelial cells (blue squares). (**D**) The team then reprogrammed the NG2 and SOX6 positive progenitors to produce corticospinal neurons. This involved antagonizing the expression of *Olig2* to prevent the cells from generating oligodendrocytes, and transiently activating *Neurog2* to initiate the program that differentiates progenitors into glutamatergic neurons (represented as a circle cell body transitioning into a triangle). The gene *Fezf2* was then expressed to promote the cells to develop into subcortical projection neurons with long axons. (**E**) The reprogrammed cells then underwent various experiments, including electrophysiology (to measure neuronal activity), RNA-seq (to see which genes are expressed), and immunocytochemistry (to see which protein markers are present). This revealed that the reprogrammed cells exhibited cardinal features of corticospinal neurons, with no detectable hallmarks of alternative fates.

Image credit: Alfonso Aguilera.

Next, Ozkan, Padmanabhan et al. designed a protocol to stop the precursors from producing oligodendrocytes and instead develop into corticospinal neurons. These neurons are a type of subcortical projection neuron that reside deep within the cerebral cortex, and have long range axons that send the neurotransmitter glutamate to nerves in the spinal cord to control voluntary movements. They are also severely affected in the neurodegenerative disease Amyotrophic Lateral Sclerosis (ALS).

After purification, the team induced and repressed the expression of various proteins in the progenitor cells to faithfully reproduce the developmental trajectory of corticospinal neurons during embryogenesis (Figure 1D). In brief, an artificially designed molecule capable of antagonizing the expression of *Olig2* was added to block oligodendrocyte differentiation (Zhou et al., 2001). The gene *Neurog2* was then transiently activated to induce the differentiation program for glutamatergic neurons (Nehme et al., 2018; Péron et al., 2023), and *Fezf2* was overexpressed to drive the specification of subcortical projection neurons (Tsyporin et al., 2021; Galazo et al., 2023).

Ozkan, Padmanabhan et al. then performed in-depth longitudinal analyses to evaluate the success rate of their reprogramming procedure. They found that, over time, the newly generated neurons exhibited molecular and functional hallmarks of corticospinal neurons, with no detectable features associated with alternative neuron fates (Figure 1E).

This study builds on previous evidence showing that activating fate-specific genes can push subsets of cells towards alternative identities and connectivity. Interestingly, Ozkan, Padmanabhan et al. achieve higher specificity for corticospinal neuron reprogramming than previous studies, in which a significant fraction of reprogrammed cells retained fate markers associated with the original identity of the cells (De la Rossa et al., 2013; Felske et al., 2023; Rouaux and Arlotta, 2013). Still, it is worth noting that these earlier studies conducted their reprogramming procedures in vivo, which provides more insight into how well a process would work as a potential treatment.

In the future, it will be exciting to see whether the in vitro protocol created by Ozkan, Padmanabhan et al. can be applied in vivo. If so, it will also be interesting to see how well the reprogrammed corticospinal neurons integrate and function in both physiological conditions and animal models of neurodegenerative diseases. The work of Ozkan, Padmanabhan et al., and other recent studies (Marichal et al., 2023), are setting the foundations for ‘a la carte’ recipes of molecules that can forge specific reprogrammed neurons with the goal to repair the brain.
